# Supplementary material for: Ultrafast light field tomography for snapshot transient and non-line-of-sight imaging
Source: Nat Commun. 2021 Apr 12;12:2179. doi: 10.1038/s41467-021-22461-0 (PMC8041853; doi:10.1038/s41467-021-22461-0)
Supplement: Supplementary file 1 — Supplementary Information [file 41467_2021_22461_MOESM1_ESM.pdf]

Supplementary Materials for
Ultrafast light field tomography for snapshot transient and non-line-of-sight imaging

Xiaohua Feng[1], Liang Gao[1,2,3*]
[1]Department of Bioengineering, University of California, Los Angeles, 90064, USA.
[2]Department of Electrical and Computer Engineering, University of Illinois at Urbana-Champaign, 61801, USA
[3]Beckman Institute for Advanced Science and Technology, 405 N. Mathews Ave., University of Illinois at Urbana-Champaign, 61801, USA.
Correspondence: Liang Gao, Email: gaol@ucla.edu

Supplementary Note 1. Light field tomography (LIFT) image formation

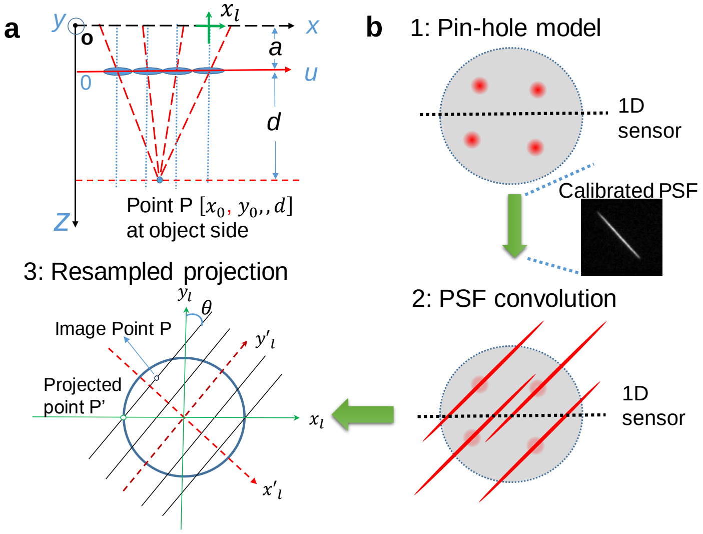

Supplementary Figure 1: Image formation modeling of LIFT. a, Two-plane parameterization of light field. **b,** Image formation of LIFT modeled by a three-step decomposition. The inset depicts an experimentally calibrated PSF for a cylindrical lenslet, showing a small vignette at the extreme ends of the PSF.

We analyze in detail the working principle of LIFT here. Although relay systems are usually added for different applications, light field acquisition by a cylindrical lenslet array remains the same for all embodiments. We use two-plane parameterization for light field analysis and do not consider occlusions here. For clarity, only four lenslets are shown in Supplementary Figure 1a, where the spatial axis (x) coincides with the sensor plane, and the angular axis (u) resides on the lenslet-array plane. Each lenslet is also assigned with a local coordinate x_l (in green), whose origin is the image of a point source located at infinity (indicated by the dashed blue lines).

The image formation onto a 1D sensor by a cylindrical lenslet is artificially decomposed into three steps here: (1) pin-hole image formation, (2) PSF substitution, and (3) resampled projection.

Step 1: pin-hole image formation model.
This is the classical imaging process. Consider a point source located at $[x_0, y_0, d]$, the pin-hole model predicts its local coordinates on a sub-image as:

$$\begin{cases} x_l = \frac{a}{d}(u - x_0) & (a) \\ y_l = -\frac{a}{d}y_0 & (b) \end{cases}, \quad (1)$$

Step 2: PSF convolution
A cylindrical lenslet differs from a perfect spherical lens in lacking optical power along one axis, which we referred to as the invariant axis. For a point source, it forms a finite line along the invariant axis at the image plane. The line length is determined by the image magnification $m = d/a$ of the system and the lenslet size as $l = (1 + 1/m)q$, where q is the lenslet diameter. Such a line-shaped PSF disperses each point in the image space onto a pixel on a 1D sensor, as illustrated by the transition from step 1 to step 2 in Supplementary Figure 1b. Therefore, an individual pixel integrates the image along the PSF-line, and a parallel beam projection of the image is obtained on the 1D sensor along the angle of the invariant axis.

Step 3: Resampled projection
For a fixed 1D sensor, the projection along different angles is acquired by rotating the cylindrical lenslet. As a result, the 1D sensor is generally not perpendicular to the projection direction. This is illustrated in step 3 of Supplementary Figure 1b, where solid black lines indicate the projection direction and the green x_l axis represents the 1D sensor. To relate the unknown image to the acquired projection data via Fourier slice theorem, it is necessary to make the projection perpendicular to the 1D sensor. This can be done by a computational resampling process. Denoting the angle between the projection and the y_l axis as θ, one can establish a local coordinate $[x_l', {y'}_l]$, shown in red dashed lines, to obtain a virtual sensor line x_l' that is perpendicular to the projection direction. These two local coordinates are related by a rotation matrix:

$$\begin{bmatrix} x' \\ y' \end{bmatrix} = R_\theta \begin{bmatrix} x \\ y \end{bmatrix} = \begin{bmatrix} cos\theta & -sin\theta \\ sin\theta & cos\theta \end{bmatrix} \begin{bmatrix} x \\ y \end{bmatrix}. \quad (2)$$

Combining Supplementary Equation (1) and (2), the image point in the auxiliary coordinate system is obtained as:

$$\begin{cases} x_l' = \frac{1}{m}(u - x_0)\, cos\theta - \frac{1}{m}y_0 sin\theta & (a) \\ y_l' = \frac{1}{m}y_0 sin\theta + \frac{1}{m}y_0 cos\theta & (b) \end{cases}. \quad (3)$$

The projection onto the virtual line sensor is done by simply dropping the *y* component:

$$\begin{cases} x_l' = \frac{1}{m}[-x_0 - y_0 tan\theta + u]cos\theta & (a) \\ y_l' = 0 & (b) \end{cases}. \quad (4)$$

Substituting the result back into Supplementary Equation (2), the experimentally recorded projection data is obtained as $x_l = {x'}_l/cos\theta$. We dub the $cos\theta$ term as the **resampling factor**: it resamples the experimentally recorded projection data (on the sensor line x_l) onto the desired recording line ${x'}_l$. In other words, each cylindrical lenslet performs a **resampled projection** onto the 1D sensor x_l. Ultimately, the LIFT imaging acquisition can be summarized into a single equation:

$${x'}_l = \frac{1}{m}[-x_0 - y_0 tan\theta + u]cos\theta. \quad (5)$$

The first two terms describe the projection process and the third term is the light field component contributed by different lenslets, which enables post-capture refocusing and depth retrieval as discussed in Supplementary Note 3.

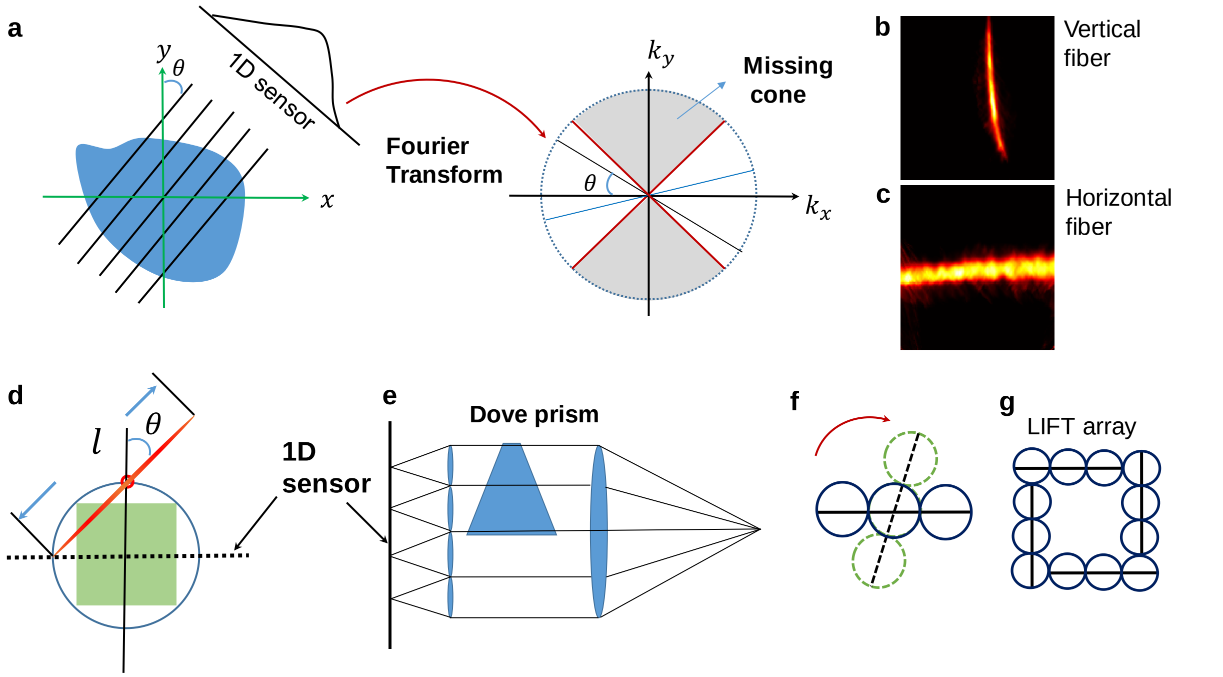

Supplementary Figure 2. LIFT data sampling analysis in the Fourier domain. a, Illustration for Fourier slice theorem and the limited view problem. **b-c**, Experimental images captured by the current LIFT camera for a fiber oriented at horizontal and vertical directions. **d,** Projection angle and field of view tradeoff for recording projection data at different angles on a 1D sensor by rotating a cylindrical lenslet. **e,** LIFT implementation using a Dove prism to span the projection angular range to [0, 180], eliminating the limited view problem. Four lenslets are shown for illustration purposes. **f-g,** Rotation of a LIFT camera or a LIFT camera array eliminates the limited view problem and enriches the number of projections and light field data. Only three lenslet are shown in the 1D camera for simplicity.

Supplementary Note 2. LIFT sampling requirement and limited view problem

2.1 Sampling: The Fourier slice theorem[1] is illustrated in Supplementary Figure 2a: the Fourier transform of the resampled projection is a slice of the two dimension Fourier transform (*k-space*) of the original image. For image reconstruction, therefore, it is necessary to fill the complete *k-space* by acquiring projection data at a sufficient number of angles spanning the range of [0°, 180°]. A rule of thumb for this criterion states that to reconstruct an $N \times N$ image, N projections with ~ N pixels resolution are needed. Using 1D sensors with a limited pixel count (several thousands) for an image resolution over 100 × 100, practical implementation of LIFT usually restricts the number of projections on the order of ten. This casts LIFT as a sparse view CT problem. Using n lenslets, the compression factor in LIFT for sampling an $N \times N$ image is therefore N/n, which is on the order of ten for most implementations. To minimize the correlations in the projection data in LIFT and therefore maximize information content for reconstruction, it is also beneficial to arrange the projection angle uniformly.

2.2 Limited view Problem. With a 1D sensor being fixed, the practical angular range of projection is also limited if only rotating the cylindrical lenslet. This is illustrated in Supplementary Figure 2b, where the line-shaped PSF is finite in length (l as predicted in Supplementary Note 1). The maximum height for a point detectable by the 1D sensor is thence limited to $h = lcos\theta/2$. This implies the achievable field of view is $2h = \ lcos\theta$. As a result, one must strike a balance between the FOV and angular range. In practical implementations, the angular range is limited to $[\theta_1, \theta_2]$, leading to a missing cone in the *k-space* as indicated by the gray area in Supplementary Figure 2a. Tomographic reconstruction in this case results in degraded image quality, which is referred to as the limited view problem. Our current LIFT implementation suffers from a limited view problem since the angular range of projection is about [-45°, 45°] with respect to the y axis. Supplementary Figure 2b-c show respectively two experimentally acquired images of a fiber oriented at vertical and horizontal directions: due to the limited view problem, the horizontal fiber shows about 2~3 times lower resolution.

2.3 Remedy limited view problem. There are several methods for mitigating this problem. One way resorts to deep learning: by training a neural network for the system with enough data that is afflicted by the limited view problem, the network can learn the pattern (or statistical distribution) of imperfections in the reconstructed image and corrects them thereafter. This solution is system-specific and can substantially mitigate, but not eliminate, the limited view problem. The second method is to insert a Dove prism after a relay lens, which projects the image of the original object to infinity, as diagrammed in Supplementary Figure 2e. The Dove prism is rotated by 45 degrees so that the image passing through it is rotated by 90 degrees, allowing the cylindrical lenslet behind it to fill in the missing cone and thus eliminating the limited view problem. The downside of using a Dove prism is that it introduces astigmatism for non-collimated light and chromatic aberrations for broadband scenes, compromising the 3D imaging performance of LIFT. Another practical method is to rotate the camera or equivalently, build a camera array as shown in Supplementary Figure 2f-g. This requires the camera to be compact and, if using rotation, the intended applications to be repeatable, such as NLOS imaging using compact SPAD cameras. For instance, rotating a LIFT camera with 7 lenslets by 3 times will not only enrich the projections to 21 for eliminating the limited view problem but also extend the light field to 2D. A similar gain can be obtained by camera array implementation. Because the deep learning method has the advantage of simplicity and faster image reconstruction, it is the method of choice for current demonstration.

Supplementary Note 3. LIFT light field imaging capabilities

3.1 Refocusing. As depicted in Supplementary Figure 3a, to focus on a different plane d_2, the 1D sensor needs to be moved by Δa. The light field at the new virtual sensor plane is calculated as:

$$x_{l2} = \left(1 + \frac{\Delta a}{a}\right) x_l - \frac{\Delta a}{a} u = \left(1 + \frac{\Delta a}{a}\right) [x_l + su], \qquad (6)$$

where $s = \ -\Delta a/(\Delta a + a)$. Ignoring the magnification factor $\left(1 + \frac{\Delta a}{a}\right)$, which is constant across the whole image area when computationally refocusing, one can rewrite Supplementary Equation (6) as:

$$x_{l2} = x_l + su. \qquad (7)$$

This is exactly the same refocusing formula in the ray space for light field cameras[2] except that LIFT captures only the angular information along one axis (u) instead of two. Hence, refocusing onto different depths can be achieved in LIFT by shearing-and-reconstructing: shear the acquired

projection data and then perform image reconstruction. This is also clear from Supplementary Equation (5), which describes the light field at plane d when the nominal focal plane is at infinity.

Two points on light field characteristic of LIFT are in order. The first is on the dimensionality of the angular information. While the angular information can be extended to 2D as shown in Supplementary Note 2, our current implementation records a 1D light field: there is no angular component (or disparity) along the other axis. However, LIFT still produces a 2D, rather than the 1D blurring effect in conventional 1D light field cameras (inset of Supplementary Figure 3b). This is attributed to the tomographic reconstruction nature of LIFT. We illustrate in Supplementary Figure 3b the back-projection reconstruction of a point source with three projection data. For the in-focus point, the three projections intersect at a single point and reconstruct the point correctly. When the projection data is sheared to refocus on a different plane, the three back-projected data intersect on three points that spread on a 2D area instead of falling on the same line (otherwise, the three projection directions are collinear). The second point is the generation of ghost images when an image part is heavily defocused. Due to the sparse-view acquisition, the three points in Supplementary Figure 3b are clustered together for small defocus but get well separated under heavy defocus. With more projections (views), there will be more intersection points that ultimately fill the voids in between, producing a blurring bokeh as in conventional photography. This is validated by a simulation of LIFT imaging of a point source using 7 and 127 views under heavy defocus (7 pixels disk size for a 128×128 image), where the convergence of ghost parts to a defocus blur is clearly observed. Such a behavior is not peculiar to LIFT: light field cameras with low angular resolution will also produce ghost images when refocusing far away from an image's actual focal plane[3]. A 2D angular information will benefit LIFT with an enhanced 2D reconstruction as it yields a larger number of projections and, consequently, improve 3D reconstruction as well.

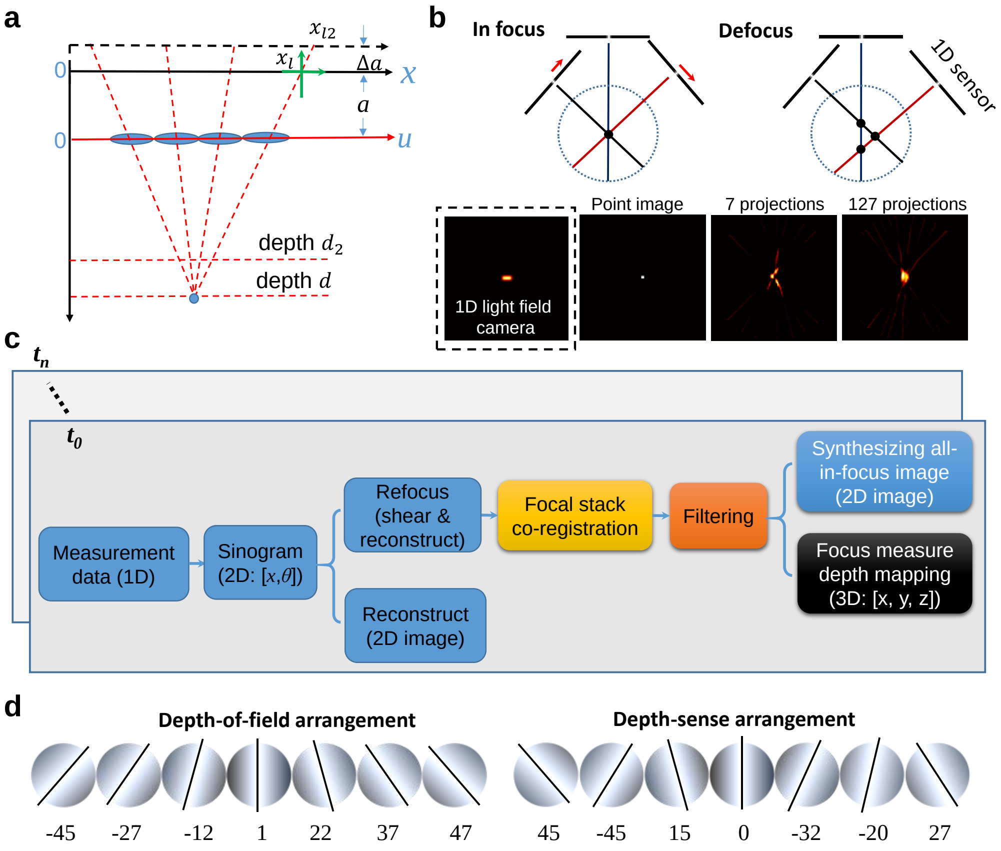

Supplementary Figure 3. **Light field imaging process for LIFT. a,** Light field propagation through different planes for refocusing. **b,** Illustration of ghost image generation in LIFT reconstruction. Defocus leads to ghost image generation in sparse view tomographic reconstruction. However, with more views, it eventually converges to a blurring bokeh as in conventional imaging methods. **c,** Processing pipeline for 2D (x, y) and 3D (x, y, z) imaging in LIFT. For 4D (x, y, z, t) imaging, the 3D image processing is individually applied at each time instance. **d,** Experimental lenslet arrangement for the depth-of-field and depth-sense version of LIFT, with black solid line representing the invariant axis of the cylindrical lenslet. The angles underneath each lenslet is w.r.t the y axis (counterclockwise being the positive direction) and listed with an accuracy of 1 degree for clarity.

3.2 Computationally extending depth of field. To extend the depth of field in LIFT, the measurement data is processed to reconstruct an image set computationally refocused on all different depths. Next, the sharpest feature around a region of interest (ROI) for each pixel is identified across the image set, and an all-in-focus image is subsequently assembled by combining the sharpest parts via graph cut algorithms[4]. Such an extended depth of field is obtained at the expense of processing time. Also, it requires the image to show enough features. The depth-of-field version of LIFT described below sidesteps these two drawbacks all together.

3.3 Lenslet arrangement. LIFT can achieve an extended depth of field without resorting to computational refocusing: by arranging the cylindrical lenslet judiciously, an all-in-focus image can be automatically obtained. Adding a shearing term to Supplementary Equation (5) for refocusing, one obtains:

$$\frac{x'_l}{\cos\theta} = -\frac{1}{m}[x_0 + y_0 \tan\theta - u] + su$$

$$= -\frac{1}{m}[x_0 - u] - \frac{1}{m}[y_0 tan\theta - msu]$$
$$= -\frac{1}{m}[x_0 - u] - \frac{1}{m}\left[y_0 - \frac{m}{tan\theta}su\right] tan\theta$$
$$= -\frac{1}{m}[x_0 - u] - \frac{1}{m}[y_0 - \Delta y] tan\theta \ , \quad (8)$$

where $\Delta y = \frac{m}{tan\theta}su$ is the shift in the object space due to refocusing. Notably, there is an interaction between the lenslet projection angle θ and the angular component u of the light field. If the cylindrical lenslets are arranged in such a manner that the angle θ as a function of the angular u axis satisfying:

$$tan\theta \ \approx \ ku, \quad (9)$$

then $\Delta y = \frac{m}{k}s$ will be a constant independent of the angular variable u, meaning no blurring in the resultant image because all lenslets contribute the same shift for a specific object plane (indexed by s). The object plane at different depths is indeed shifted from the nominal center by an amount determined by its defocus distance, but each plane is well focused. This makes the reconstructed image automatically all-in-focus. This configuration is dubbed as the **depth-of-field version** (Supplementary Figure 3d). Still, it is feasible to undo this automatic all-in-focus imaging effect and computationally defocus by changing the shearing term s from a constant to a lenslet-specific number $s \times tan\theta$.

The cost of this arrangement is a degraded depth retrieval accuracy because only residual defocus errors are left, which are contributed by the approximation error in Supplementary Equation (9). To optimize lenslet configuration for 3D imaging, we expand $tan\theta$ as a function of u into a Taylor series:

$$tan\theta \ = f(u) = m_1 u + m_2 u^2 + \ \dots \ . \quad (10)$$

Substituting into Δy, it becomes evident that maximizing the first-order coefficient m_1 leads to the depth-of-field lenslet configuration, whereas minimizing it to zero will maximize the defocus error and hence optimize depth retrieval. It is straightforward to perform a search over the permutations of pre-determined set of projection angles to find the near-optimum configuration for depth retrieval. The resultant configuration is dubbed as the **depth-sense version** (Supplementary Figure 3d).

Supplementary Figure 4 demonstrates experimentally that refocusing in the depth-of-field version of LIFT camera only induces an image shift. When computationally sweeping the focus from near (a) to far (d), the reconstructed helical fiber is shifted upwards from left to right. However, the helical fiber structure is well resolved in all the cases despite of its large depth range.

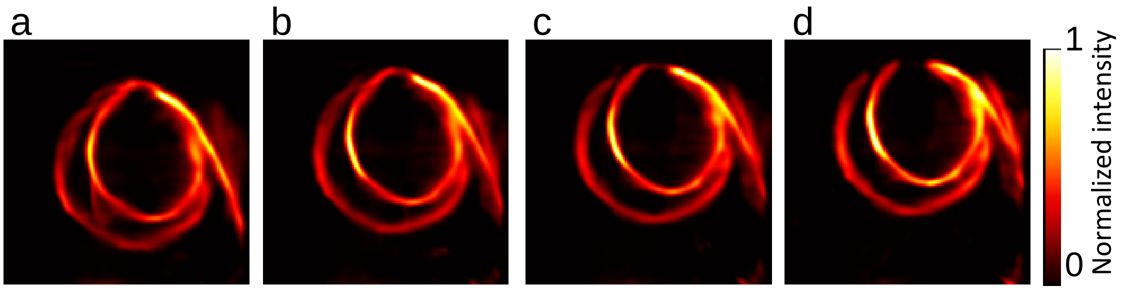

Supplementary Figure 4. Automatically extended depth of field via lenslet arrangement. a-d, The image is refocused at different depths from near to far. Except for a shift in the *y* direction, the image is almost identical. The last image falls slightly out of FOV, cropping its top parts.

3.4 Depth retrieval. LIFT can extract depths via the depth-from-focus (DfF) method[5], thereby yielding a 3D image at each time instance. In DfF, the camera captures a sequence of images of the scene at different focal settings, producing a focal stack. To infer depth, a focus measure (sum of modified Laplacian[6]) is computed for each pixel of the image, and the focal setting giving rise to the maximum focus measure is identified, which can be mapped to a depth value. For light field cameras like LIFT, the focal stack is captured with a single snapshot and produced computationally by refocusing the image at different depths.

The processing pipeline of LIFT for reconstructing multi-dimensional images (2D, 3D, and 4D) is summarized in Supplementary Figure 3c. Each 1D measurement data is ordered into a sinogram (the projection data (x, θ)), which can be directly reconstructed into a 2D *(x, y)* image or go through a shear-and-reconstruct process to refocus on different depths, producing a focal stack. Afterwards, the focal stack is co-registered because the refocusing can induce image shifts, as explained in the previous Supplementary Note. The denoising algorithm VBM3D is then applied to attenuate the refocusing artefacts in the focal stack, which substantially improves the robustness of depth retrieval. Finally, the focus measure is computed for each pixel, and a quick sorting algorithm identifies the correct focal setting and map that pixel to the corresponding depth, yielding the 3D image *(x, y, z)*. Owing to the decoupled space-time acquisition in LIFT, the 2D and 3D images processing are independently performed at each time instance to produce the final 3D *(x, y, t)* or 4D *(x, y, z, t)* results.

The focus-to-depth mapping for LIFT is illustrated in Supplementary Figure 5a, where the image relay system is not included. A relay system with a magnification of M changes the depth retrieval accuracy by M^2. For a plane at $[0, 0, d]$, the distance between the leftmost and rightmost sub-images is:

$$L = \frac{d+a}{d} D \, , \qquad (11)$$

where D is the baseline length of the lenslet array and a is its distance to the sensor. To connect depth d with refocusing parameter s, it is noted that the distance L at infinity is $L_\infty = D$ and refocusing from infinity onto depth d involves shearing the light field, which leads to $L_\infty = L + s(u_l - u_r) = L + sD$, where u_l and u_r indicates the leftmost and rightmost angular components, respectively. Solving above equation yields $d = \frac{a}{s}$.

The depth retrieval accuracy Δd is the minimum depth change that causes a one-pixel variation in the distance L. Given a linear sensor with N_x pixels across the baseline, a one-pixel change is $\Delta L = D/N_x$. Taking the derivative of Supplementary Equation (11) with respect to *d*, one obtains $\Delta d = \frac{d^2}{Da}\Delta L = \frac{d^2}{N_x a} = \frac{m^2 a}{N_x}$. As a equals approximately to the lenslet focal length f, the depth retrieval accuracy can be estimated as $\Delta d = \frac{m^2 f}{N_x}$.

Supplementary Figure 5b-e demonstrated an example of 3D imaging of a slanted plane that displays a grid of points. With an imaging magnification ~18, a focal length of 8 mm and N_x ~1200, Δd is estimated to be ~2 mm, which agrees well with the inferred value from the calibration curve.

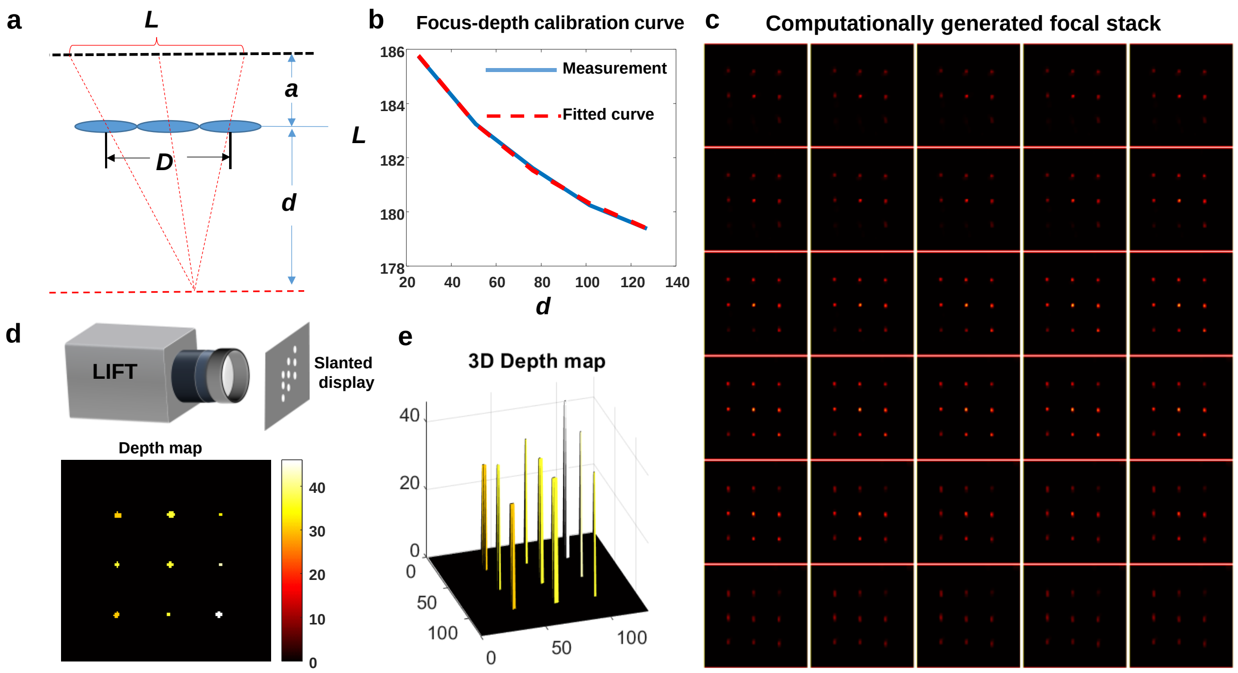

Supplementary Figure 5. LIFT depth-retrieval. a, Schematic for LIFT camera depth retrieval accuracy analysis. **b,** Calibrated *d*-*L* curve for an imaging magnification ~18. Fitting data with Supplementary Equation (11) can extract parameters *a* and *D*. **c-e**, Computationally generated focal stack, extracted depth map, and 3D rendering of a slanted plane displaying a grid pattern of dots. The slanted shape of the plane is well reproduced. It is also noted that defocus causes the dots to become dimmer in the focal stack as energy gets spread onto a larger area.

Supplementary Note 4. Modeling of non-ideal effects in LIFT system

In practical system implementations, there will be some misalignments between the 1D sensor and the individual cylindrical lenslets, which will affect image quality if not accounted for. As the misalignment of a lenslet shifts the image from its ideal position by a vector $\vec{r}$, it can be modeled as a convolution operation with a shifted Dirac delta function $\delta(x - \vec{r})$. The forward model in LIFT can then incorporate the non-ideal effect as:

$$y = ABx = Ax', \tag{12}$$

where x' is the uncorrected image vector, and B is the convolutional matrix:

$$B = \begin{bmatrix} P_1 \\ P_2 \\ \vdots \\ P_n \end{bmatrix}, \tag{13}$$

with P_i being the block Toeplitz matrix of point spread function of lenslet i. By calibrating with an arbitrary point source, indicated as vector e_k, one can reconstruct the point spread function of the non-ideal system as $x' = PSF' = Be_k$, which recovers the matrix B. The true image x can then be recovered by deconvolving x' with the calibrated PSF' using the Richard-Lucy algorithm.

Supplementary Figure 6 illustrates experimentally the benefits of modeling non-ideal effect in LIFT. The calibrated point spread function and the iteratively reconstructed image of the helical fiber without and with applying deconvolution are shown in Supplementary Figure 6a-c respectively. Supplementary Figure 6d-e depict the DANN reconstruction results. In both iterative

and DANN reconstructions, the image resolution is improved by approximately two folds after deconvolution, despite of some amplified noises.

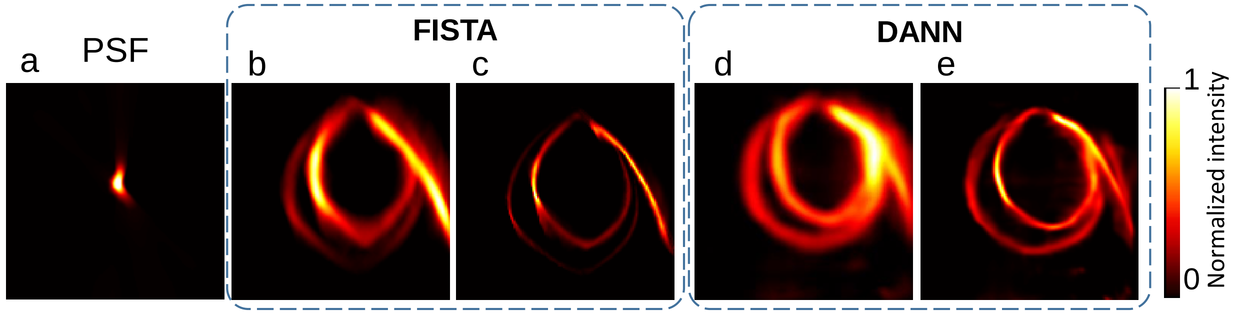

Supplementary Figure 6. Modeling non-ideal effects for image reconstruction. a, Calibrated system point spread function. **b** and **c**: reconstructed images of the helical fiber without and with deconvolution for the iterative methods using FISTA algorithm. **d** and **e** reconstructed images of the helical fiber without and with deconvolution by DANN.

Supplementary Note 5. LIFT imaging quality

The image quality of LIFT depends on both the compression factor, or equivalently, the number of projections, and the signal to noise ratio of the measurement data.

5.1 Compression factor. The sampling analysis in the previous Supplementary Note indicates that a larger number of projections will fill the *k-space* more densely and therefore lead to better reconstruction quality in LIFT due to the reduced compression factor. This is illustrated in Supplementary Figure 7, which shows the recovered images for a Shepp-Logan phantom and a cluttered camera-man photograph using different number of projections at a resolution of 128×128. The sampling angular range is [0°, 180°] and the transform function $\varphi(g)$ is chosen as total variation (TV) to encourage sparsity in image gradient. Sampled at the Nyquist rate, the images recovered with a projection number of 128 serves as the ground truth reference for calculating the peak signal to noise ratio (PSNR) of other reconstructed images. It is noted that, as the compression factor gets larger (i.e., fewer projections), the PSNR of the reconstructed images becomes smaller and fine image details gradually get washed out. Moreover, the cluttered camera-man photograph renders a smaller PSNR than that of the Shepp-Logan phantom when employing the same compression factor. Therefore, the number of projections must be appropriately scaled to accommodate scenes of different complexity. This is expected and conforms to the general observations in sparse view CT reconstruction.

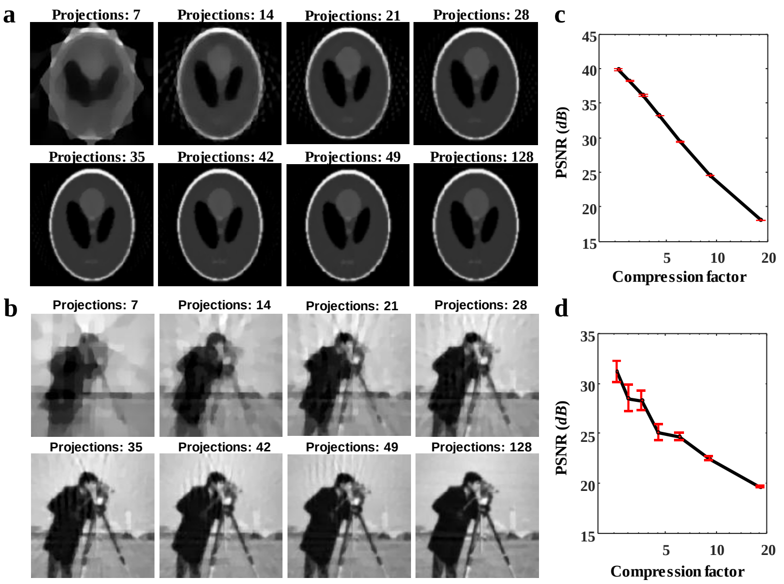

Supplementary Figure 7. LIFT image reconstruction using different number of projections for **a,** Shepp-Logan phantom and **b,** the cluttered camera-man photograph, both at a resolution of 128×128. The compression factor varies from ~18 to 1 (Nyquist rate) when the projection number changes from 7 to 128. **c** and **d**, The PSNR of the reconstructed images versus the compression factor (1 not included as it corresponds to the reference image) for the phantom and camera-man photograph, respectively.

5.2 Noise robustness. As ultrafast imaging with picoseconds resolution is usually shot-noise limited, we study the noise robustness of LIFT by varying the average number of photons (K) in the recorded projection data. The images are then reconstructed using 7 projections spanning an angular range of [0°, 180°] by the FISTA algorithm. For comparison, the ground truth images are also simulated using the same average number of photons K in the image. Supplementary Figure 8a-b shows, respectively, the reconstructed and ground truth images of the Shepp-Logan phantom when the average number of photons varies from 4 to 256. With a few photons, fine details are generally masked out even in the ground truth images, and only a rough structure of the image can be recovered by LIFT. However, with the photon count reaching over 100, the recovered image in LIFT begins to converge to the ideal reconstruction results.

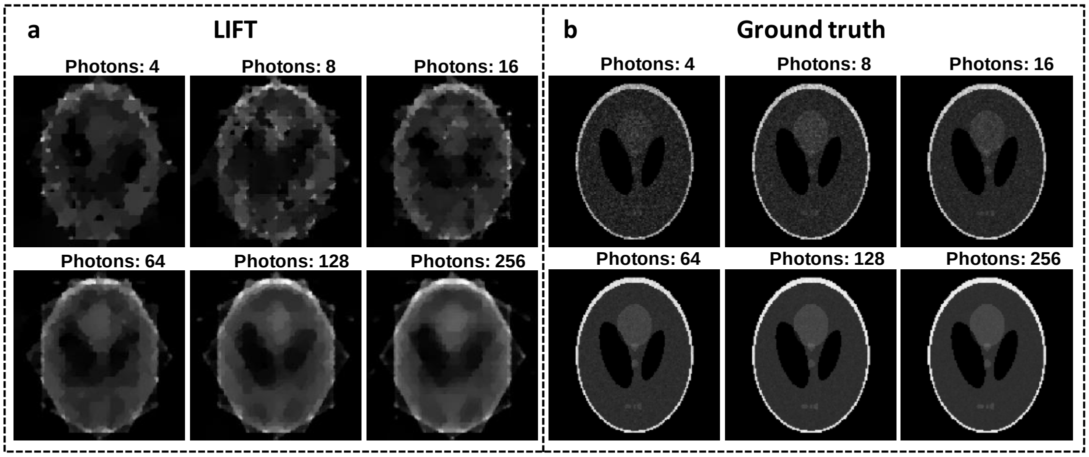

Supplementary Figure 8. LIFT image reconstruction under different noise levels. a, Reconstructed images by LIFT. **b,** Ground truth images.

Both data fidelity and regularization terms in the optimization-based formulation contribute to the improved noise robustness for LIFT reconstruction over filtered backprojection. The data fidelity is a least square term that tends to suppress noises at the expense of resolution. The regularization term is critical for noise attenuation as it denoises intermediate reconstructions in each iteration, which is particularly evident under the framework of regularization by denoising (RED) for inverse problems.

Supplementary Note 6. NLOS imaging via LIFT

6.1 Experimental setup. We summarize below the equipments used in LIFT system.

1. Streak camera: C13410-01A (Hamamatsu Photonics), 10000:1 dynamic imaging range, effective image resolution: 1314 (slit direction) × 1016 (time direction), frame rate: 100 Hz (storing or transferring). Observation window: variable from 500 ps to 1 millisecond long.
2. Cylindrical lenslet: plano-convex, custom-made, 2 mm diameter, 8 mm focal length.
3. Ultrafast photodiode: 818-BB-45 (Newport Inc.), 500 nm~ 890 nm, rise time ~30 ps.
4. Picosecond laser: Spark Sirius (Spark-Lasers Inc.), 532 nm, 6 ps pulse width, 2 mW average power at 100 Hz repetition rate.
5. Video camera: Hero4 Silver (GoPro Inc.), 1080p at 60 Hz maximum.

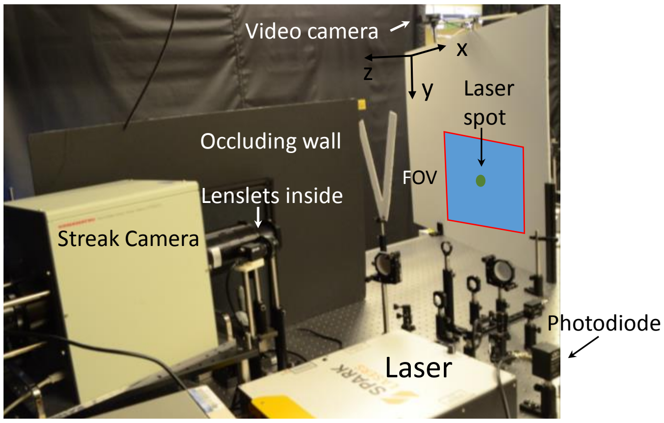

Supplementary Figure 9. NLOS imaging experimental setup. A picosecond laser is collimated onto the central region of the diffusing wall. The FOV on the wall is about 600 mm × 800 mm for both static and dynamic NLOS imaging. The observation time window is adjusted for imaging at different scales.

6.2 Compressibility of the NLOS (x, y, t) datacube. Mediated by a relay wall, NLOS imaging shows drastically different characteristics from natural photographs: the instantaneous (and steady state) images on the wall are generally smooth and highly compressible, even for complex hidden scenes. We show the instantaneous images on the wall for hidden scenes of different complexity, which were simulated by a transient render[7] on publicly accessible datasets[8]. For these synthetic datasets, the time bin is 10 ps, and the laser incident point is at the center of the wall. The camera (point scanning or parallel detectors) samples the wall at a resolution fixed at 128×128, regardless of the grid size. To simulate the recoverable *(x, y ,t)* datacube by the LIFT camera, we employed a two-step process: 1) changing the PSF of the camera in the synthetic dataset to line-shaped PSFs to model the LIFT camera, and 2) reconstructing the datacube by the iterative FISTA algorithm, owing to its greater flexibility to handle LIFT models using different number of projections. As NLOS imaging typically employs compact SPAD sensors and does not require to record the

complete *(x, y ,t)* datacube with a single laser shot, we synthesize the LIFT camera model *A* to encompass up to tens projections that sample uniformly the complete angular range of [0°, 180°]. This can be easily achieved by a few rotations of a 1D SPAD based LIFT camera as discussed in Supplementary Figure 2d. The hidden scenes are reconstructed at a volumetric resolution of 128×128×128 using the extended phasor-field method.

Supplementary Figure 10a depicts the instantaneous images on the wall at several representative time instants for a hidden resolution target, which is placed 0.5 m away from the wall (grid size: 1 m × 1 m). The recovered instantaneous images using different number of projections are shown in the second to fourth row. The *(x, t)* slices at different *y* of the datacube and reconstructed hidden scene (maximum intensity projection along the depth direction) are compared in a tabular format in Supplementary Figure 10b and c, respectively. It is noted that the instantaneous images are highly structured and smooth. As a result, LIFT can recover well both the instantaneous images and the hidden scene using only 14 projections, corresponding to ~10% of the data load in the point-scanning method. Compared with the ground truth images, LIFT results tend to be smoother, particularly for the cases using a small number of projections. This is attributed to the LIFT's radial sampling pattern in *k*-space: high spatial frequencies are more sparsely sampled, as indicated in Supplementary Figure 2a. Nonetheless, LIFT using 7 to 14 projections can still detect, though not resolve, the smallest strips in the resolution target. The reconstruction by LIFT using 7 projections rendered the main shapes of the resolution target despite of some artefacts.

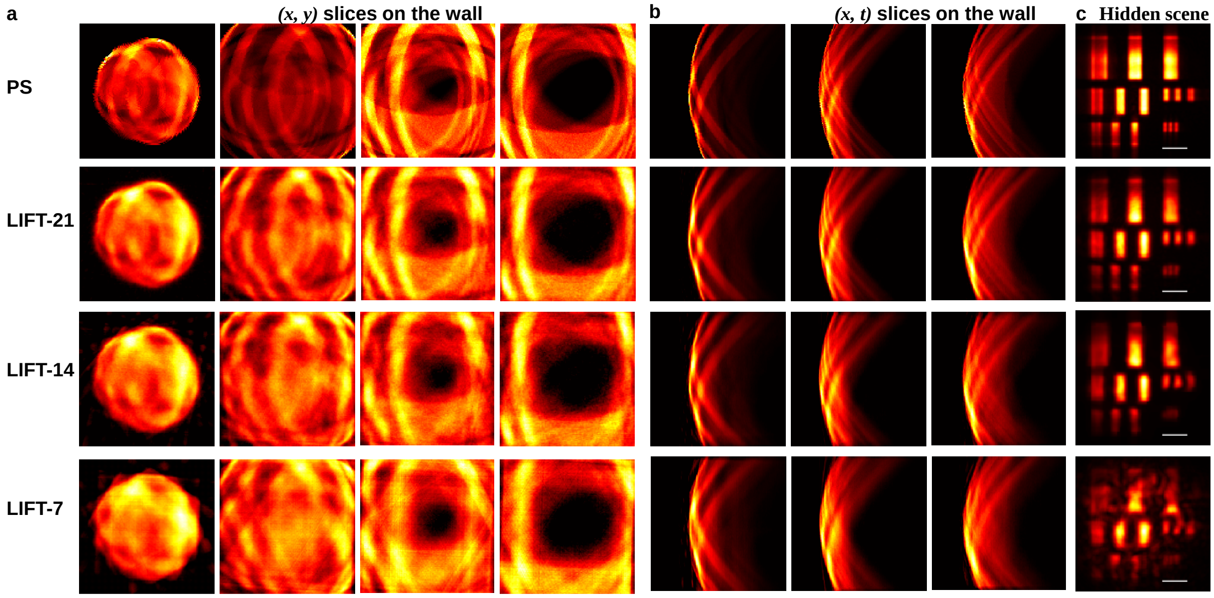

Supplementary Figure 10. Compressibility of NLOS imaging for the hidden scenes of a resolution target. **a,** *(x, y)* images— the instantaneous images on the wall, acquired by point-scanning (ground truth) and the LIFT camera using different number of projections. **b,** *(x, t)* slices at different *y* of the corresponding spatiotemporal data cube on the wall. **c,** The reconstructed hidden scene. PS: point-scanning data acquisition. LIFT-*N*: LIFT data acquisition using *N* projections. Scale bar: 140 mm.

Supplementary Figure 11 shows the results for a complex bookshelf scene ~2 m away from the wall (grid size 1.8 m × 1.8 m). The same observations can be made: LIFT using only 14 projections can recover the hidden scene decently although there is a slight resolution degradation and an increase in artefacts/noises. Using 7 projections (compression factor ~20) for data acquisition, LIFT can recover the main shapes of the scene despite of some background noises.

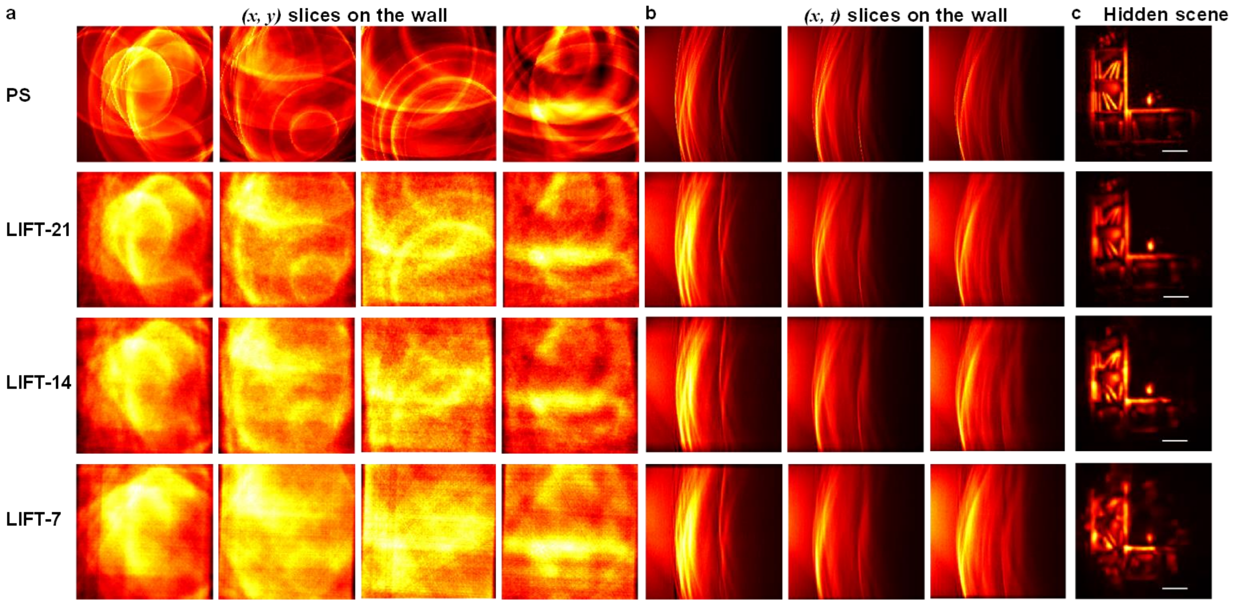

Supplementary Figure 11. Compressibility of NLOS imaging for the complex bookshelf scenes. **a,** The *(x, y)* images on the wall at different time instants. **b** *(x, t)* slice of the spatiotemporal data cube at different *y* on the wall. **c,** The reconstructed hidden scene. PS: point scanning data acquisition. LIFT-N: LIFT data acquisition using N projections. Scale bar: 280 mm.

6.3 Noise robustness. The robustness to noises was studied for the bookshelf scene that suffers from strong inter-reflections. The globally maximum photon count in the data cube is varied as in previous works[9]. For LIFT, the maximum photon counts are in the projection measurement rather than the reconstructed *(x, y, t)* datacube. Supplementary Figure 12a shows, in a tabular arrangement, the reconstructed bookshelf using a maximum photon count ranging from 2000 to only 50 along the row direction and a varying number of projections in the column dimension. The reconstruction results by the phasor-field method using point-scanning are given in Supplementary Figure 12b as references, whose maximum photon counts are varied from 200 to 5. While the point-scanning method recovers the bookshelf with a maximum photon count of 10, LIFT using 21 projections need 100 counts to recover the main shapes of the bookshelf. This indicates that LIFT using 21 projections is about 10 times nosier than the point-scanning method. Less projections in LIFT requires more photons to recover the hidden scene and tends to produce smoother results. This is expected as less projections will produce stronger reconstruction artefacts and noises.

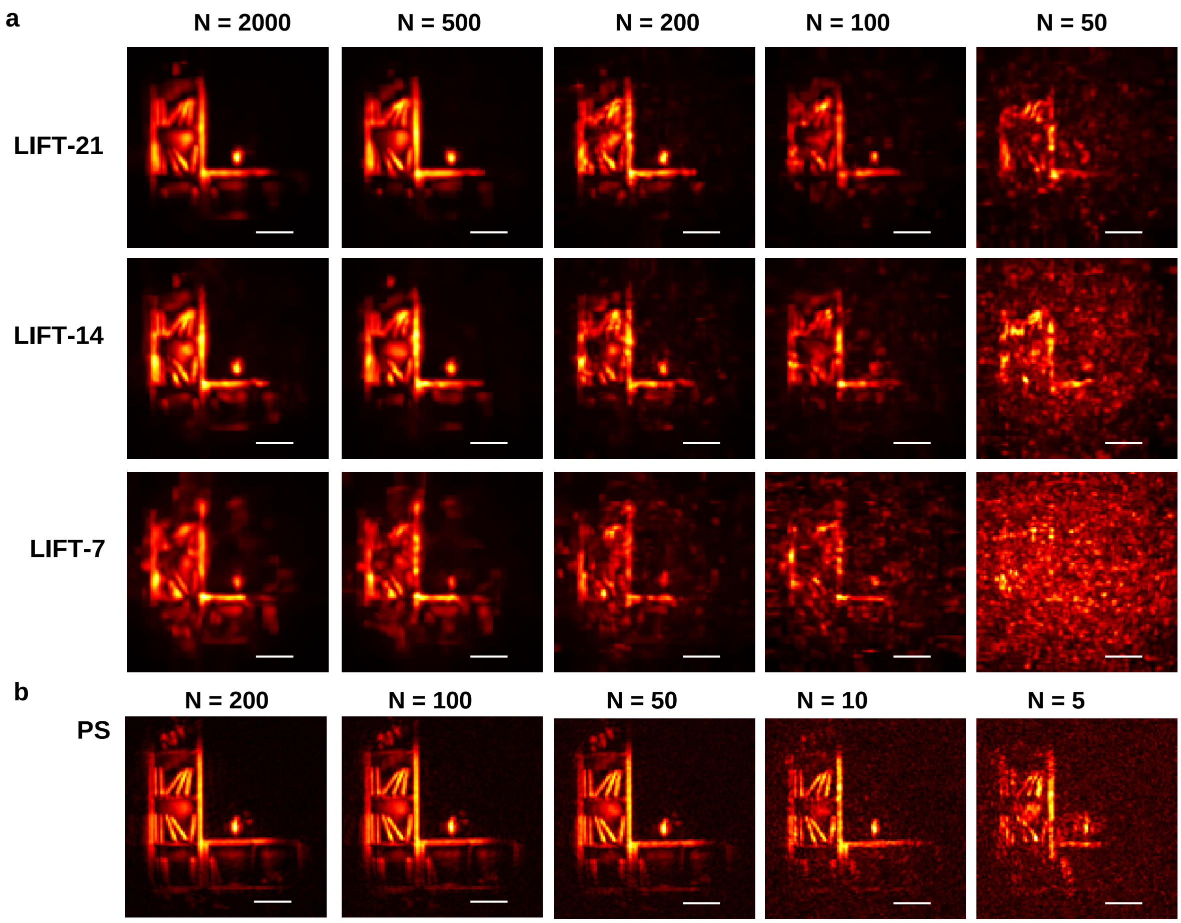

Supplementary Figure 12. Noise robustness of LIFT camera for NLOS imaging of a complex hidden scene. **a,** reconstruction of the bookshelf by LIFT using various photon counts and different number of projections, **b,** reconstruction of the bookshelf by the point scanning method using different photon counts. Scale bar: 280 mm.

However, LIFT can readily compensate for its noisier reconstruction by allowing longer exposure time while still maintaining 30 Hz video rate. With 7 projections in a 1D SPAD camera, LIFT can acquire 21 projections using only three rotations, leading to an exposure time of 10 ms at each rotation for imaging at 30 Hz. In contrast, point scanning[10] at 32×32 resolution is still ~ ten times away from 30 Hz, even using an exposure time as short as ~250 μs. Scanning a 1D SPAD array along one spatial axis can reach 30 Hz at a resolution of 100×100 but only at an exposure time of 300 μs (30 ms/100) for each line, which is 30 times shorter than that of LIFT. Compared with 2D SPAD cameras, LIFT using 1D SPAD array benefits from ~10 times larger fill factor, which currently floats around 10% in state-of-the-art 2D designs. Therefore, LIFT can collect over ten times more photons to compensate for its higher noise level while offering unique advantages: compressive data acquisition and full-fledged light field capabilities. Given an *(x, y, t)* datacube of 128×128×1000, acquired with 8 bit precision, the resultant data load is 16 Megabytes, more than twice of that in 4K ultra high definition camera. Streaming such data at 30 Hz reliably typically requires nontrivial compression algorithms. Instead, LIFT with 21 projections reduced the data load during acquisition more than six times. Moreover, the light field capability of LIFT is inherently challenging to implement in scanning-based methods or 2D SPAD cameras without incurring a substantial increase in system complexity and data load.

Lastly, we summarize the photon counts used in the simulation. For the bookshelf scene, the time bin is 10 ps, and the global maximum photon count is 100 for LIFT reconstruction, leading to an average of ~20 photons per time bin in the window of [0, 24 ns]. This corresponds to reconstruction in the point-scanning method using a maximum of 10 photons (1.2 average photons per bin in the same time window). After accounting for the time bin difference, this is on par with previous experimental data of a complex hidden scene that has a maximum of 6 photons with a 4 ps time bin, acquired using 1 ms exposure[9]. This validates that LIFT using 1D SPAD cameras holds promise for large scale NLOS imaging at 30 Hz video rate.

6.4 Resolution of NLOS imaging. The resolution of NLOS imaging is primarily determined by the camera temporal resolution τ (with system jitter if temporal scanning/averaging is employed) and wall size w[11] . For current NLOS demonstration, the temporal resolution τ (jitter-limited) is about 60 ps, leading to an axial resolution of $\Delta z = \frac{c\tau}{2} \cong 9\ \text{mm}$. The theoretical lateral resolution is derived by O'Toole et al.[11] as $\Delta x = c\tau \frac{\sqrt{(w/2)^2+z^2}}{w}$, with z being the distance of the hidden scene to the wall. A similar resolution bound is also found by Liu et al.[9] However, LIFT reconstruction through either iterative methods or deep adjoint neural network does not achieve a perfect recovery of the ground truth images, particularly for the high-frequency details as shown in Supplementary Figure 10-11. Also, the image reconstruction of LIFT tends to render worse resolution in scenarios with noisy measurement data (see Supplementary Note 5). These factors degraded the lateral resolution of LIFT for NLOS imaging in practice.

Supplementary Figure 13 shows the characterization of the NLOS imaging resolution using measurement data with a low SNR. The hidden scene consists of two 20 mm wide strips separated by 100 mm, placed ~ 250 mm away from the wall. The raw temporal signals with maximum intensity in each lenslet (Supplementary Figure 13a) show large noises, causing the reconstruction to suffer from artefacts and a worse resolution. As 20 mm is smaller than practical lateral resolution, the strip image renders the line spread function of the system. The resolution is then estimated to be ~40 mm by averaging the two strips' full width half magnitude (FWHM) in Supplementary Figure 13c. The theoretical lateral resolution is ~18 mm in this case, with an effective wall size (containing NLOS signals) being ~400 mm while the camera FOV on the wall is ~600 mm. The resolution can be improved by using more projections (by camera rotation or camera array) and a higher laser power for an improved SNR.

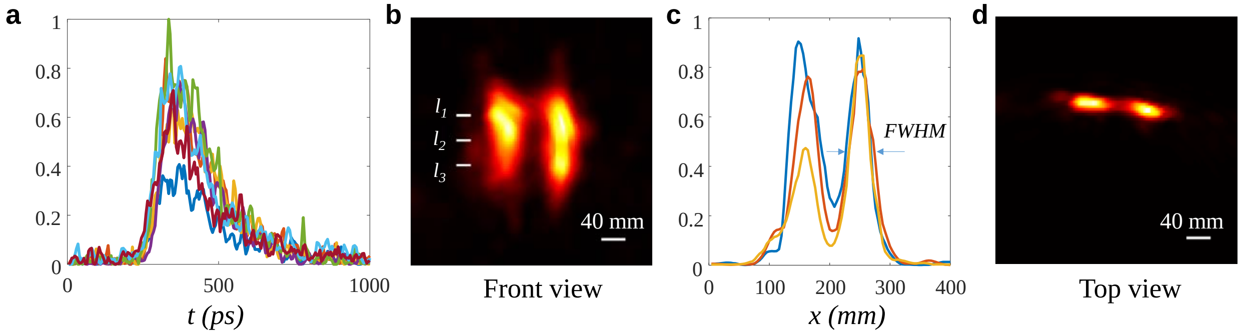

Supplementary Figure 13. NLOS imaging resolution with LIFT. **a,** Maximum measured temporal signal in the sub-image of each lenslet. The signal is advanced here with respect to the true time zero. Note that all the signals show large variances, indicating relatively low signal to noise ratios. **b,** Maximum intensity projection of the reconstructed two strips. **c**, Line profiles of the image as indicated in **b**. The resolution is estimated by the FWHM. **d,** Top view of the reconstruction.

Supplementary Note 7. Benchmark LIFT against state-of-the-art transient and NLOS imaging methods

To better understand the strengths and limitations of LIFT for transient imaging in general and NLOS imaging in particular, we compare it with other ultrafast cameras (Supplementary Table 1) and NLOS imaging methods (Supplementary Table 2) below. It is noted that, given the same signal to noise ratio, image acquisition at/over the Nyquist sampling rate generally represents an upper bound on the imaging quality (contrast, spatiotemporal resolution), a condition that compressive imaging methods asymptotically approach. While LIFT image acquisition is scalable in the compression factor and can attain up to the Nyquist sampling rate (as discussed in Supplementary Note 2.2), its practical implementations mostly fall into the compressive regime. As a result, we only compare the imaging metrics based on our current compressive LIFT camera.

Supplementary Table 1 Comparison of transient imaging performance by various methods

Methods	Resolution	Temporal resolution	Sequence depth	Compression factor	Light field	Active illumination	Scanning
STEAM[12]	50×50	> 10 ns	Continuous	NA	No	Yes	No
STAMP[13]	450×450	~ 230 fs	6	NA	No	Yes	No
FRAME[14]	512×512	~ 125 fs	4	NA	No	Yes	No
CUP[15]	150×150	~ 10 ps	350	~ 100	No	No	No
LIFT	128×128	< 10 ps	> 1000	~ 18	Yes	No	No
SPAD[16]	320×240	~ 300 ps	~ 300	NA	No	No	Yes (64 s)
SPAD[17]	256×250	~ 300 ps	> 1000	NA	No	No	Yes (1 s)

For NLOS imaging, the comparison excludes imaging metrics obtained using retro-reflective objects since they are less common and typically render orders of magnitude stronger signals than diffusive targets. Also, the camera's spatial resolution on the wall is in lieu of 100×100 (except for the edge-resolved transient imaging (ERTI) that only involves a one-dimensional angular scanning). Since the spatial resolution of NLOS imaging degrades linearly with the distance to the wall, the listed resolution is accompanied with the distance at which it was evaluated. One notable exception is ERTI, which has a constant angular resolution that makes its lateral resolution, which equals to the angular resolution times the distance to the wall, degrades at a faster rate as in phase array radar imaging.

LIFT features unique light field capability with the deepest sequence yet manages to use a small compression factor for snapshot 2D transient imaging with a resolution over 120×120. While SPAD cameras[16,17] can acquire high-resolution images at the Nyquist rate and, therefore, accommodate cluttered natural scenes better, the need of spatial scanning and repeated illuminations leads to prolonged acquisition. Interestingly, the transient images at each time instant obtained by SPAD cameras[16,17] also show notable compressibility—they are far simpler than the static photograph of the cluttered scene, which will be accentuated with a higher temporal resolution. The snapshot acquisition enables LIFT to achieve drastically faster NLOS imaging with a resolution and quality close to those in dense point-scanning methods, allowing a low laser power to be used for imaging over 1 m scale. By scaling according to the r^4 photon decay law in NLOS imaging, LIFT is expected to reach an imaging volume around 3 m × 3 m × 3 m with an average laser power of 160 mW. Its light field capabilities will also be an important ingredient towards translating NLOS imaging to field deployment.

Supplementary Table 2 Benchmark of NLOS imaging performance

Methods	Resolution	Speed	Quality	Power	Scale	Light field	Full 3D imaging
Confocal point scanning[10]	20 mm (0.4 m)	> 20 s to hours	Excellent	1 W	~ 3 m	No	Yes
Non-confocal point scanning[9]	20 mm (0.4 m)	> 20 s to hours	Excellent	1 W	~ 3 m	No	Yes
ERTI[18]	2.4~4.8 degrees	> 900 s	Good	120 mW	~ 3 m	NA	No
LIFT	~ 40 mm (~0.3 m), scalable	Snapshot to 1 s	Good ~ excellent	2 mW	~ 1.2 m	Yes	Yes

Supplementary Note 8. Deep adjoint neural network reconstruction

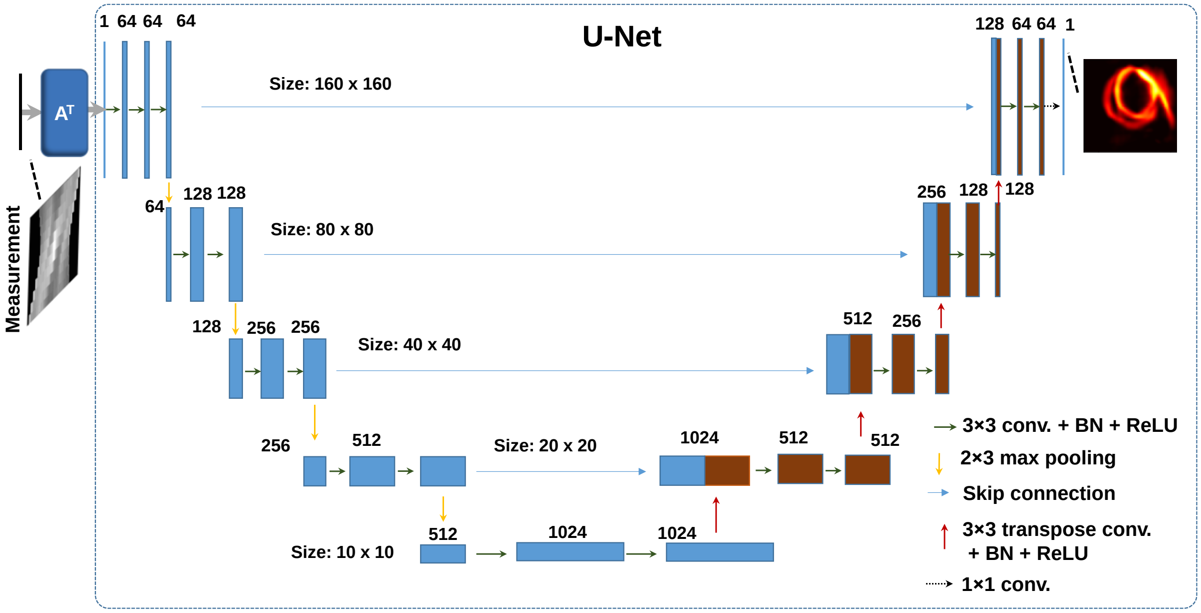

Supplementary Figure 14. Deep adjoint neural network structure for LIFT image reconstruction. The acquired LIFT sensor data is arranged into a sinogram and passed to the system adjoint operator A^T before being fed to the deep neural network, which is a U-net[19] with skip connections[20,21]. For training data acquisition, the LIFT camera captures (without temporal deflection) the training images streamed on a high-resolution monitor in a synchronized manner. The whole training dataset contains 48000 images selected from MNIST and FashionMNIST datasets and it took about three hours to complete acquisition. To test the reconstruction performance of the DANN network, a test dataset consisting of 1000 images (not contained in the training dataset) was created from the two datasets as well. It is noted that the test dataset is also composed with images afflicted with limited view problem. The DANN network was trained with Adam algorithm for five epochs with a batch size of 16. **conv.**: convolution; **BN**: batch normalization; **ReLU**: rectified linear unit.

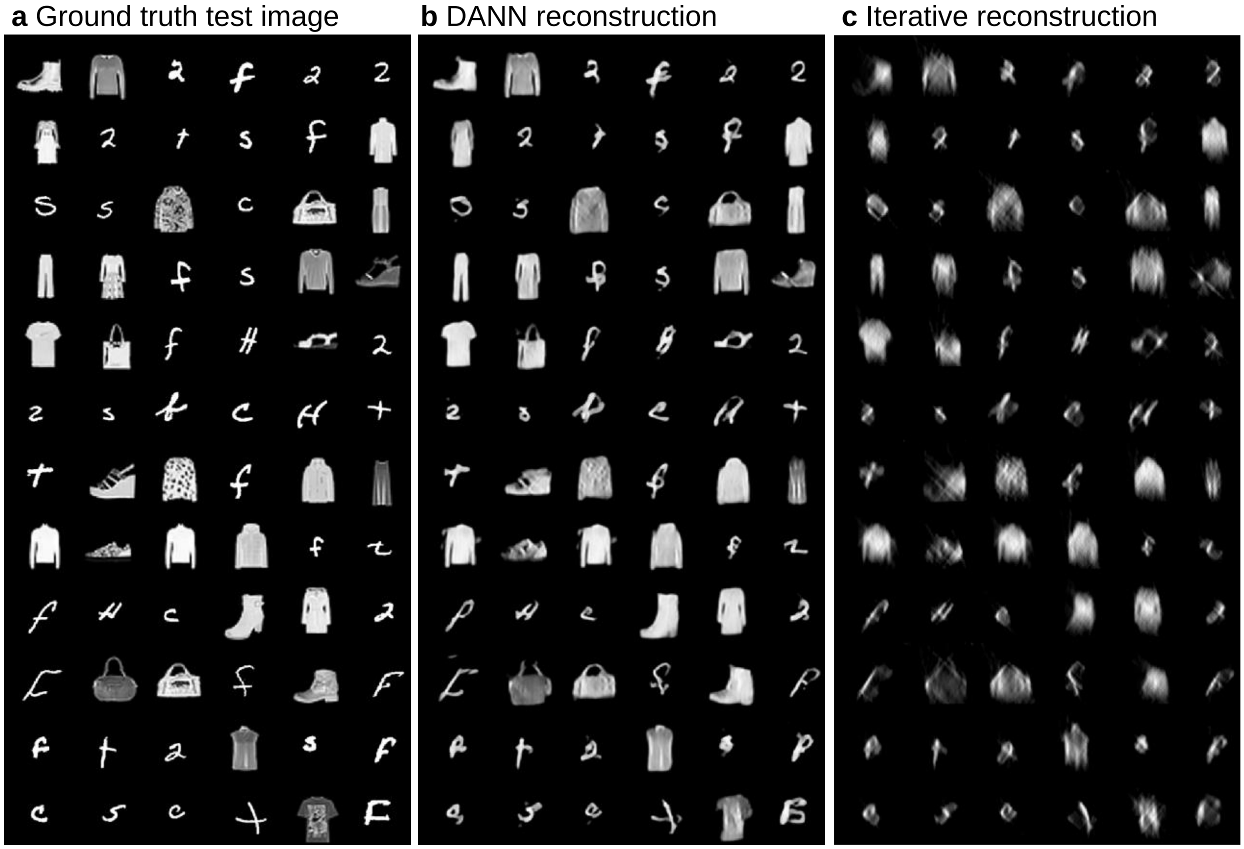

Supplementary Figure 15. Reconstruction results of a test data set afflicted by limited view problem by DANN and iterative methods. a, ground truth image. **b** and **c**, DANN and iterative reconstruction respectively. The DANN reconstruction recovers the image with better fidelity and significantly less limited view problem, which manifest as dimmer signals and lower resolution in the iterative reconstruction results.

Supplementary References

1. Kak, A. C. & Slaney, M. *Principles of Computerized Tomographic Imaging*. (Society for Industrial and Applied Mathematics, 2001). doi:10.1137/1.9780898719277.
2. Ng, R. DIGITAL LIGHT FIELD PHOTOGRAPHY. 203 (Stanford University, 2006).
3. Wu, G. *et al.* Light Field Image Processing: An Overview. *IEEE Journal of Selected Topics in Signal Processing* **11**, 926–954 (2017).
4. Boykov, Y., Veksler, O. & Zabih, R. Fast Approximate Energy Minimization via Graph Cuts. *IEEE TRANSACTIONS ON PATTERN ANALYSIS AND MACHINE INTELLIGENCE* **23**, 18 (2001).
5. Schechner, Y. Y. & Kiryati, N. Depth from defocus vs. stereo: how different really are they? in *Proceedings. Fourteenth International Conference on Pattern Recognition (Cat. No.98EX170)* vol. 2 1784–1786 (IEEE Comput. Soc, 1998).
6. Nayar, S. K. & Nakagawa, Y. Shape from focus. *IEEE Transactions on Pattern Analysis and Machine Intelligence* **16**, 824–831 (1994).

7. Jarabo, A. *et al.* A framework for transient rendering. *ACM Trans. Graph.* **33**, 177:1-177:10 (2014).
8. Galindo, M. *et al.* A dataset for benchmarking time-resolved non-line-of-sight imaging. in *ACM SIGGRAPH 2019 Posters* 1–2 (Association for Computing Machinery, 2019). doi:10.1145/3306214.3338583.
9. Liu, X. *et al.* Non-line-of-sight imaging using phasor-field virtual wave optics. *Nature* **572**, 620–623 (2019).
10. Lindell, D. B., Wetzstein, G. & O'Toole, M. Wave-based Non-line-of-sight Imaging Using Fast F-k Migration. *ACM Trans. Graph.* **38**, 116:1-116:13 (2019).
11. O'Toole, M., Lindell, D. B. & Wetzstein, G. Confocal non-line-of-sight imaging based on the light-cone transform. *Nature* **555**, 338–341 (2018).
12. Goda, K., Tsia, K. K. & Jalali, B. Serial time-encoded amplified imaging for real-time observation of fast dynamic phenomena. *Nature* **458**, 1145–1149 (2009).
13. Nakagawa, K. *et al.* Sequentially timed all-optical mapping photography (STAMP). *Nature Photonics* **8**, 695–700 (2014).
14. Ehn, A. *et al.* FRAME: femtosecond videography for atomic and molecular dynamics. *Light: Science & Applications* **6**, e17045–e17045 (2017).
15. Gao, L., Liang, J., Li, C. & Wang, L. V. Single-shot compressed ultrafast photography at one hundred billion frames per second. *Nature* **516**, 74–77 (2014).
16. O'Toole, M. *et al.* Reconstructing Transient Images from Single-Photon Sensors. in *2017 IEEE Conference on Computer Vision and Pattern Recognition (CVPR)* 2289–2297 (2017). doi:10.1109/CVPR.2017.246.
17. Lindell, D. B., O'Toole, M. & Wetzstein, G. Towards transient imaging at interactive rates with single-photon detectors. in *2018 IEEE International Conference on Computational Photography (ICCP)* 1–8 (2018). doi:10.1109/ICCPHOT.2018.8368466.
18. Rapp, J. *et al.* Seeing around corners with edge-resolved transient imaging. *Nature Communications* **11**, 5929 (2020).
19. Ronneberger, O., Fischer, P. & Brox, T. U-Net: Convolutional Networks for Biomedical Image Segmentation. *arXiv:1505.04597 [cs]* (2015).
20. He, K., Zhang, X., Ren, S. & Sun, J. Deep Residual Learning for Image Recognition. in *2016 IEEE Conference on Computer Vision and Pattern Recognition (CVPR)* 770–778 (IEEE, 2016). doi:10.1109/CVPR.2016.90.
21. Jin, K. H., McCann, M. T., Froustey, E. & Unser, M. Deep Convolutional Neural Network for Inverse Problems in Imaging. *IEEE Transactions on Image Processing* **26**, 4509–4522 (2017).
