# Supplementary material for: Ultrafast light field tomography for snapshot transient and non-line-of-sight imaging
Source: Nat Commun. 2021 Apr 12;12:2179. doi: 10.1038/s41467-021-22461-0 (PMC8041853; doi:10.1038/s41467-021-22461-0)
Supplement: Supplementary file 3 — Description of Additional Supplementary Files [file 41467_2021_22461_MOESM3_ESM.pdf]

Description of Additional Supplementary Files

File name: Supplementary Movie 1
Description: 3D imaging of a picosecond laser pulse propagation inside a fiber.

File name: Supplementary Movie 2
Description: Deep adjoint neural network for mitigating the limited view problem.

File name: Supplementary Movie 3
Description: Animated 3D visualization of Letters V and I.

File name: Supplementary Movie 4
Description: Video rate NLOS imaging at 30 Hz.
